# Social functions of relaxed open-mouth display in golden snub-nosed monkeys (*Rhinopithecus roxellana*)

**DOI:** 10.24272/j.issn.2095-8137.2018.043

**Published:** 2018-08-13

**Authors:** You-Ji Zhang, Yi-Xin Chen, Hao-Chun Chen, Yuan Chen, Hui Yao, Wan-Ji Yang, Xiang-Dong Ruan, Zuo-Fu Xiang

**Affiliations:** 1 Institute of Evolutionary Ecology and Conservation Biology, Central South University of Forestry & Technology, Changsha Hunan 410004, China; 2 College of Life Science and Technology, Central South University of Forestry and Technology, Changsha Hunan 410004, China; 3 Shennongjia National Park, Shennongjia Forest District, Shennongjia Hubei 442411, China; 4 National Forest Inventory and Design Institute, Beijing 100714, China

**Keywords:** Golden snub-nosed monkey, Open-mouth, Submission, Reconciliation, Affiliation, Reassurance

## Abstract

Relaxed open-mouth display serves important social functions in relation to submission, reconciliation, affiliation and reassurance among non-human primate societies; however, quantitative evidence on this behavior remains insufficient among multi-level social groups. From July to November 2016, we examined four potential functions of the relaxed open-mouth display during pairwise, intra-unit social interactions among 18 free-ranging adult and sub-adult golden snub-nosed monkeys (*Rhinopithecus roxellana*) who belonged to three one-male, multi-female units (OMU) at Dalongtan, Shennongjia National Park, China. Results showed that: compared with no relaxed open-mouth display, (1) the occurrence of displacement by a dominant individual approaching a subordinate was lower and the distance of the subordinate to the approaching dominant was shorter when the subordinate showed open-mouth display; (2) relaxed open-mouth display reduced the probability of continued attack for victims of aggression and allowed victims to achieve closer proximity to the aggressor during post-conflict periods; (3) relaxed open-mouth display by dominant individuals allowed them to achieve closer proximity to subordinates; and (4) the exchange of relaxed open-mouth display had a greater impact on the outcome of interactions than one individual alone giving this signal. These findings suggest that relaxed open-mouth display serves important functions regarding submission, reconciliation, affiliation and reassurance in coordinating social interactions within OMUs in golden snub-nosed monkeys.

## INTRODUCTION

Open-mouth display is a common social behavior in non-human primates. It comprises individuals opening their mouths and presenting their teeth or canines and can be associated with different body postures and behaviors in either hostile or relaxed scenarios (Altmann, 1962; Hinde & Rowell, 1962; Yang et al., 2013). There are diverse patterns and functions of open-mouth display among primates. In hostile scenarios, open-mouth displays are aggressive. As observed in *Macaca mulatta* and *Macaca arctoides*, open-mouth display by dominant individuals involves the slight opening of mouths and presentation of mandibles, followed by teeth chattering, pulling back of lips and orienting their heads towards subordinates while making threatening calls; if subordinates do not respond submissively, the aggression can escalate to ritualized fighting or physical attack, resulting in subordinate displacement (Altmann, 1962; Hinde & Rowell, 1962; Ren et al., 1990a).

In addition to the functions of aggression in hostile scenarios, researchers have noted that relaxed open-mouth displays in normal, calm or post-conflict periods are crucial in many primate species for submission, reconciliation and reassurance to coordinate social interactions, increase social tolerance and maintain group stability (Aureli, 1997; de Waal, 1986; de Waal & Luttrell, 1989; de Waal & Ren, 1988).

Until now, only a handful of studies have quantitatively tested functional hypotheses of relaxed open-mouth display in behavioral interactions among non-human primate societies. For example, within social groups of *Trachypithecus francoisi*, lower-ranked members exhibit submissive behavior when being attacked by dominant individuals, including opening mouths and showing teeth, shaking heads slightly, and keeping the body and limbs slack, to ease tension and decrease the possibility of continuous attack (Wang, 2009). In groups of *Mandrillus sphinx*, frequencies of relaxed open-mouth display and teeth chattering during post-conflict periods between two individuals are significantly higher than during pre-conflict periods, suggesting that relaxed open-mouth display may have important reconciliatory effects (Bout & Thierry, 2005). In groups of *Macaca sylvanus*, the exchange of relaxed open-mouth display (i.e., presented by both subordinate and dominant individuals) has greater impact than when one individual alone displays the signal (Wiper & Semple, 2007). Relaxed open-mouth display may also function as reassurance, a kind of affiliative signal exhibited by dominant individuals opening their mouths slightly and approaching subordinates under normal, relaxed scenarios (van Hooff, 1967; Wiper & Semple, 2007), as observed in groups of *Mandrillus sphinx* and *Macaca sylvanus* (Bout & Thierry, 2005; Wiper & Semple, 2007).

The golden snub-nosed monkey (*Rhinopithecus roxellana*) is endemic to China, inhabiting the Sichuan, Gansu, Shaanxi and Hubei provinces (Guo et al., 2007; Yao et al., 2011). This species is characterized by a multi-level society consisting of several one-male, multi-female units (OMUs), at least one all male unit (AMU), and satellite solitary males forming a large and cohesive group (Qi et al., 2014). In addition to the common function of aggression, researchers have suggested that relaxed open-mouth display in *R. roxellana* may also function in submission, reconciliation, affiliation or reassurance during normal, calm or post-conflict periods (Li et al., 2006; Ren et al., 1990b; Yan et al., 2006; Yang et al., 2013). However, these conclusions are primarily based on qualitative observations, with no current research providing quantitative evidence regarding the functions of relaxed open-mouth display in golden snub-nosed monkeys during normal, relaxed or post-conflict periods.

Here, we studied pairwise, relaxed open-mouth display in a free-ranging group of *R. roxellana* and quantitatively tested four potential functional hypotheses–i.e., submission, reconciliation, affiliation and reassurance–during normal, relaxed and post-conflict periods. We predicted that: (1) for submission, the occurrence of displacement by a dominant individual approaching a subordinate should be less frequent when the subordinate shows relaxed open-mouth display; and the risk of continued aggression by an aggressor should be reduced if the victim shows relaxed open-mouth display; (2) for affiliation, the distance from the subordinate to the approaching dominant individual should be shorter when the subordinate shows relaxed open-mouth display; (3) for reconciliation, the distance between the aggressor and victim should be closer if the victim shows relaxed open-mouth display; and (4) for reassurance, a dominant individual should be able to approach a subordinate more closely when showing relaxed open-mouth display.

## MATERIALS AND METHODS

### Study site and study group

We conducted the study at Dalongtan, Shennongjia National Park, Hubei, China (N31${}^{\circ }$29${}^{\prime }$65${}^{\prime \prime }$, E110${}^{\circ }$17${}^{\prime }$93${}^{\prime \prime }$; 2 170 m a.s.l.). The study site is comprised of highly seasonal deciduous broadleaf and conifer forest. The monthly average temperature ranges from 17.11 ${}^{\circ }$C in July to –3.51 ${}^{\circ }$C in January (Yao et al., 2011).

During the study period, the study group consisted of 76 individuals belonging to five OMUs and one AMU. Reserve staff have successfully habituated and provisioned this group since 2006, making close observation and individual identification possible (Yao et al., 2011). We were able to identify and name each individual based on distinct physical features of the body and face, e.g., body size, fur color, shape of nipples in females, shape of granulomatous flanges on sides of upper lip in males, and body deformities. We named each OMU based on the male leader’s name (Xiang et al., 2014; Yao et al., 2011; Yu et al., 2013). Reserve staff provisioned the monkeys twice daily (1130–1200 h and 1800–1830 h, UTC+8) with lichen, pine seeds, apples, carrots, peaches and peanuts. When not provisioned, the monkeys ranged freely across an area with a radius of approximately 1 km (Xiang et al., 2014). To maximize sampling efforts due to the limited period and OMUs containing insufficient adults, we chose 18 adults and sub-adults as focal individuals (4–19 years old) from the three OMUs with the greatest number of adult females (HH, XZ, and XB, Table 1) and recorded relaxed open-mouth displays during intra-unit social interactions.

**Table 1:** Aggressive and submissive behavior sampling number and rank of each individual in three focal units

Social Unit	Individual	Sex	Age	Aggressive behavior/acts	Submissive behavior/acts	Sum	Dominance index	Ranking order
HH	HH	M	Adult	37	9	46	0.94±0.03	1
	HH_2_	F	Adult	33	15	48	0.79±0.16	2
	HHE	F	Adult	12	6	18	0.56±0.18	3
	YY_1_	F	Adult	10	5	15	0.48±0.20	4
	DWB	F	Adult	4	3	7	0.36±0.20	5
	TJ	F	Adult	14	2	16	0.32±0.13	6
	AL	F	Sub-adult	3	0	3	0.06±0.04	7
XZ	XZ	M	Adult	38	24	62	0.92±0.04	1
	XB_2_	F	Adult	36	18	54	0.80±0.15	2
	XE	F	Adult	26	12	38	0.50±0.19	3
	YB	F	Adult	24	9	33	0.41±0.15	4
	XH	F	Adult	12	8	20	0.29±0.16	5
	SB	F	Sub-adult	7	0	7	0.08±0.03	6
XB	XB	M	Adult	23	13	36	0.91±0.03	1
	GG	F	Adult	17	5	22	0.67±0.20	2
	LN	F	Adult	23	5	28	0.50±0.20	3
	SS	F	Adult	15	4	19	0.34±0.22	4
	YY_2_	F	Adult	3	1	4	0.08±0.03	5
Sum				337	139	476		

M: Male; F: Female.

### Data collection

#### Dominance rank

Our study was divided into two successive periods. In the first period (22 July–31 August 2016) before systematic sampling, we used an “aggression-submission” index to calculate the dominance rank of each individual within an OMU (Martin & Bateson, 1993). We observed the study group from distances of 3–20 m from 0830 h to 1130 h and 1430 h to 1730 h (UTC+8). Using all-occurrence sampling (Altmann, 1974), we recorded every incidence of “aggression-submission” as well as the initiators, recipients, and all social behaviors related to conflict (e.g., grasping, chasing, displacing, threatening, avoiding, crouching and fleeing) involving the 18 focal individuals. Due to our focus on intra-OMU pairwise interactions and to avoid biases from multi-individual alliances within or among OMUs, we only recorded intra-OMU aggressive-submissive behaviors involving two individuals.

We defined an individual of higher rank to be dominant in each pairwise social interaction (involving two individuals only) and the lower-ranked individual to be subordinate. Similarly, when aggression occurred, we defined the one who threatened or attacked another individual to be the aggressor and the other party to be the victim. In most cases, aggressors were dominant, and victims were subordinate (Ren et al., 1990b).

During the study period, we also provisioned the 18 focal individuals with peanuts on four occasions per month to ascertain whether intra-OMU hierarchy remaining unchanged, i.e., the “nut test” (Prud’Homme, 1991; Wiper & Semple, 2007). In the test, we positioned peanuts, a highly favored food item, at a point equidistant from two individuals to invoke a dominance-based interaction and assumed the individual who approached and ate the peanut to be the dominant of the two (Prud’Homme, 1991; Wiper & Semple, 2007). To minimize bias, we repeated the nut test three times on each occasion when we were unsure of the intra-OMU hierarchy.

#### Relaxed open-mouth display and related behavior definitions

Based on the dominance rank data obtained in the first period, during the second period (1 September–4 November 2016) we used focal animal sampling (Altmann, 1974) to record relaxed open-mouth displays of each focal individual for 30 min between 0830–1130 h and 1430–1730 h per day (UTC+8).

Focal animal sampling began when one individual approached the focal individual to within 5 m or the focal individual moved toward another individual within 5 m with eye contact (Wiper & Semple, 2007). We simultaneously recorded the identities of the two individuals who approached each other, whether displacement, aggression or continued aggression occurred, the presence or absence of relaxed open-mouth display, and the closest distance between the two individuals.

**Figure 1:**
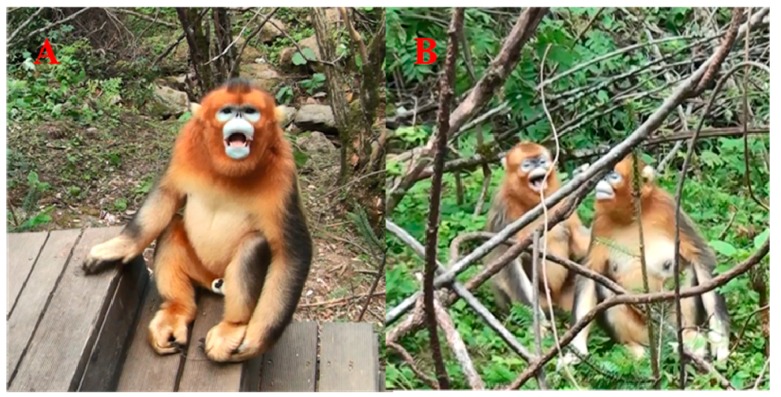
Relaxed open-mouth display in golden snub-nosed monkeys (*Rhinopithecus roxellana*) during normal, relaxed or post-conflict periods

We defined relaxed open-mouth display as an instance when an individual slightly opened mouth without presenting canines, with an associated relaxation of limbs, head, neck and shoulders (Ren et al., 1990b) (Figure 1A, B). Furthermore, during such action, the individual could approach another OMU member with only slight communicative calls rather than threatening sounds (Li et al., 1993). We considered both individuals exhibiting these actions as an “exchange of relaxed open-mouth display” (Wiper & Semple, 2007).

Canines are not presented. A: Male; B: Female. Photo by You-Ji Zhang.

**Figure 2:**
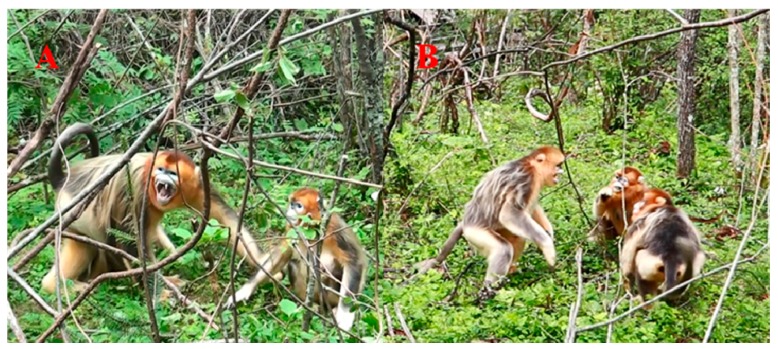
Aggressive open-mouth display in golden snub-nosed monkeys (*Rhinopithecus roxellana*) during hostile scenarios

Additionally, we defined the related behavioral patterns and hypothesized functions as follows:

Aggression: aggressors threatened and attacked victims by opening their mouths aggressively and staring, while tightening their head, neck, shoulder and limb muscles and initiating warning calls (Figure 2A, B). Ritualized fighting or physical attack could occur if victims did not respond submissively (Cui et al., 2014; Li et al., 2013).

Canines are presented, facial expressions and body postures are aggressive. A: Male; B: Female. Photo by You-Ji Zhang.

Post-conflict period: a period lasting up to 10 min after aggressors completely ceased threatening or attacking victims. During this period, relaxed open-mouth display and submissive behaviors, especially exhibited by victims, eased tension and individuals gradually returned to a relaxed or normal status (Ren et al., 1991).

Continued aggression: aggressors continued to threaten or attack victims after the initial aggression occurred, even if victims exhibited relaxed open-mouth display and submissive behaviors. Continued aggression could occur subsequently after the initial aggression or could erupt anytime within 10 min of the post-conflict period (Ren et al., 1991). It could be relentless aggressive behavior or the escalation of aggression from threat to ritualized fight, even physical attack.

Displacement: a higher-ranking individual displaced a lower-ranking individual to occupy a superior position (such as a cool, warm or feeding place). During displacement, the subordinate typically showed evasive behavior and surrendered a food resource or favored site when the dominant individual approached (Yan et al., 2006).

Submission: a series of behaviors performed by victims to prevent continued aggression, including opening their mouths and showing teeth, slightly shaking their heads, and keeping their body and limbs slack (Li et al., 2013; Wang, 2009).

Affiliation: friendly contact between individuals to maintain group balance (Ren et al., 1990b). Subordinates slightly opened their mouths or both subordinates and dominants exchanged relaxed open-mouth display to attain a closer distance and ease tension in the OMU.

Reconciliation: any affiliative contact between former opponents within 10 min after a conflict ceased (Ren et al., 1991). The function of reconciliation behavior is thought to be a recovery mechanism of the social relationship between the two opponents (de Waal & van Roosmalen, 1979).

Reassurance: dominant individuals actively and firstly opened their mouths without presenting canines to reduce the risk of aggression perceived by subordinates in post-conflict or relaxed periods. Reassurance is a form of affiliation but is considered separately as the dominant individual, rather than the subordinate, is the key signaler (Wiper & Semple, 2007).

Finally, relaxed open-mouth display in this study (Figure 1A, B) was quite different from “aggressive open-mouth display” in behavioral patterns (Figure 2A, B), although the latter is ubiquitous among *Rhinopithecus* species (*R. roxellana*: Ren et al., 1991; Yang et al., 2013; *R*. *bieti*: Li et al., 2013; *R. brelichi*: Cui et al., 2014).

### Data analysis

#### Analysis of dominance rank

We adopted the dominance index to explore the intra-OMU dominance rank of the 18 focal individuals using four steps (Ren et al., 1990c; Zumpe & Michael, 1986).

(1) Percentage of aggressive behaviors given: for each pair of individuals, we calculated the percentage of the total number of aggressive behaviors given by each participant to the other.

(2) Percentage of submissive behaviors received: for each pair of individuals, we calculated the percentage of submissive behaviors received by each participant from the other.

(3) Percentage of aggression given and submission received per pair: we combined the percentage scores of aggression given and submission received for each individual in the pair.

(4) Dominance index: we evaluated the dominance index by averaging, for each individual, the percentage scores of aggression given and submission received with all other individuals in the group.

Finally, we produced the dominance rank by sequencing the intra-OMU dominance index values from high to low.

#### Analysis of relaxed open-mouth display

We divided all behavioral data into the following categories: “absence of relaxed open-mouth display” vs. “presence of relaxed open-mouth display” and “single individual showing open-mouth display” vs. “both individuals showing open-mouth display”. We then respectively summed the occurrences of displacement and continued aggression, and distances between dominant individuals and subordinates or aggressors and victims.

We adopted the *Chi*-square test to detect differences in the occurrences of displacement and continued aggression under different relaxed open-mouth display patterns. We used one-way analysis of variance (ANOVA) for overall comparison followed by *t*-tests (SPSS v17.0) to identify differences in distances between subordinates, dominants, and/or aggressors and victims. Each sample showed normal distribution. Statistical tests were two-tailed, and significance was set at 0.05.

### Ethical statement

Prior to conducting this study, we obtained approval from the Shennongjia National Park and the Institutional Animal Care and Use Committee of Central South University of Forestry and Technology.

## RESULTS

### Dominance rank

We observed aggressive behavior 337 times and submissive behavior 139 times. Based on our calculation of dominance index, we ranked the 18 focal individuals within their OMUs as follows: HH unit, HH>HH_2_>HHE>YY_2_>DWB>TJ>AL; XZ unit, XZ>XB_2_>XE>YB>XH>SB; XB unit, XB>GG>LN>SS>YY (Table 1). The intra-OMU hierarchy remained stable throughout the entire study period (22 July–4 November 2016) based on the results from the random nut tests.

### Relaxed open-mouth display

We observed relaxed open-mouth display 212 times out of 322 total approaches within the OMUs. The occurrence of relaxed open-mouth display was related with displacement 29 times (13.68%, *n*=212), with aggression 24 times (11.32%, *n*=212), with friendliness 59 times (27.83%, *n*=212), with post-conflict period 53 times (25%, *n*=212) and with reassurance 47 times (22.17%, *n*=212). We obtained 154 h of observation on the focal group, and the average occurrence of relaxed open-mouth display for the 18 focal individuals was 1.38 times/h. The displacement occurrence of subordinates by approaching dominant individuals is shown in Table 2. There was a higher risk of being displaced by a dominant individual if the subordinate did not exhibit relaxed open-mouth display (χ^2^=25.259, *P*<0.001) (Table 2).

**Table 2:** Displacement occurrence of subordinates by approaching dominant individuals

	Occurrence of displacement event (*n*)	No. displacement event (*n*)	Statistical tests (*Chi*-square test)
Neither dominant nor subordinate showed relaxed open-mouth display	21	2	χ^2^=25.259, *P*<0.001
Subordinates alone showed relaxed open-mouth display^*^	5	24	

^*^: There were no “Dominants alone exhibited relaxed open-mouth display”.

Table 3 shows the occurrence of continued aggression during the post-conflict period. The occurrence of continued aggression was lower when victims alone exhibited relaxed open-mouth display towards aggressors (χ^2^=14.368, *P*<0.001) (Table 3).

**Table 3:** Times of continued aggression

	Occurrence of continued aggression (*n*)	No. continued aggression (*n*)	Statistical tests (Chi-square test)
Neither victim nor aggressor showed relaxed open-mouth display	10	1	χ^2^=14.368, *P*<0.001
Victims alone showed relaxed open-mouth display^*^	4	20	

*: There were no “Aggressors alone exhibited relaxed open-mouth display”.

**Figure 3:**
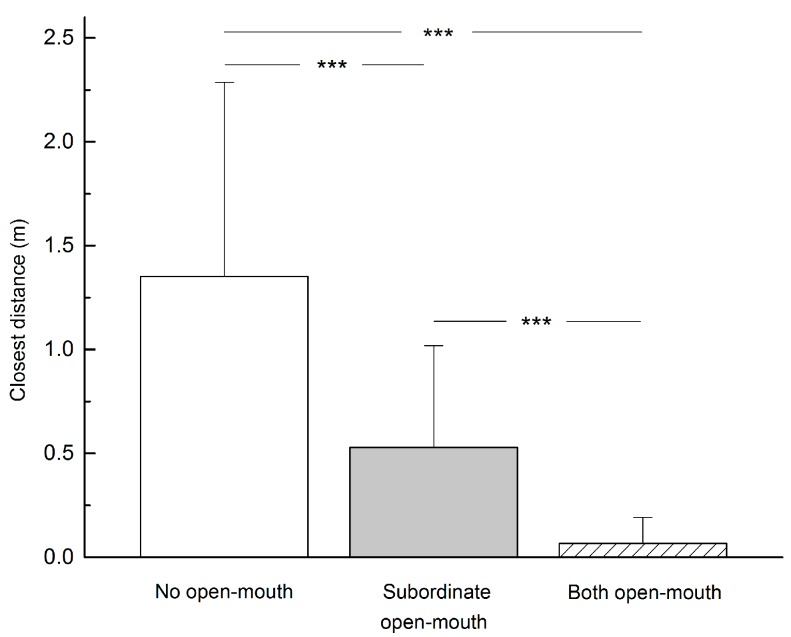
Closest distance achieved by a subordinate approaching a dominant individual (*n*=99)

We recorded three different relaxed open-mouth display patterns when subordinates approached dominant individuals, i.e., subordinates did not show open-mouth display (40 times), only subordinates showed open-mouth display (31 times) and both subordinates and dominants showed open-mouth display (28 times). The average minimum distance achieved by subordinates approaching dominant individuals without becoming the target of aggression is shown in Figure 3. Relaxed open-mouth display had a significant effect on distance (*F*=37.062, *P*<0.001). Subordinates were able to get closer to dominant individuals if they exhibited relaxed open-mouth display than if they did not (*t*=4.796, *P*<0.001). Furthermore, exchange of relaxed open-mouth display from both participants facilitated the closest distances between them (*t*=5.068, *P*<0.001).

***: *P*<0.001.

**Figure 4:**
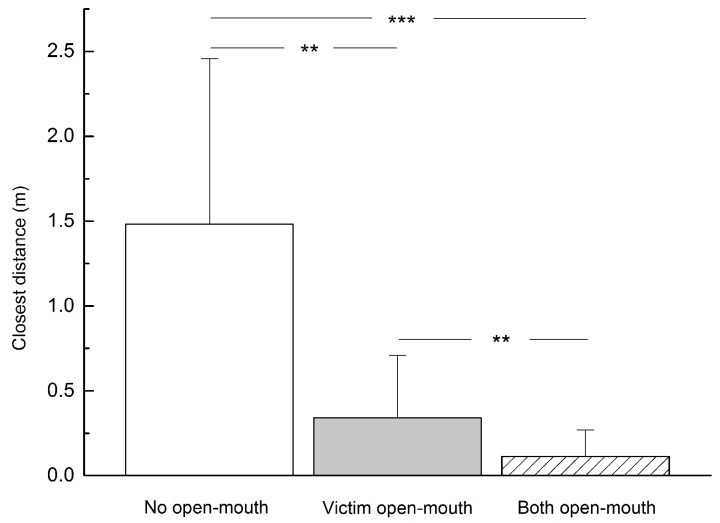
Closest distance between victim and aggressor after aggression (*n*=64)

After aggressive events ceased, we recorded three different relaxed open-mouth display patterns, i.e., victims did not show open-mouth display (11 times), victims showed open-mouth display toward aggressors (24 times) and both participants showed open-mouth display (29 times). Relaxed open-mouth display had a significant effect on distance (*F*=34.907, *P*<0.001). By exhibiting a relaxed open-mouth display, victims were able to get closer to aggressors (*t*=3.759, *P*<0.01), and exchange of relaxed open-mouth display allowed victims and aggressors to get much closer than when only victims exhibited the behavior alone (*t*=2.828, *P*<0.01) (Figure 4).

***: *P*<0.001; **: *P*<0.01.

**Figure 5:**
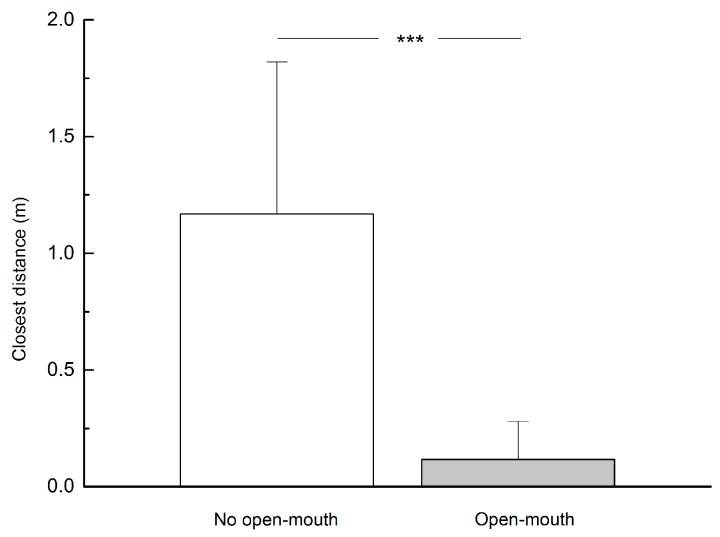
Distance achieved by a dominant individual approaching a subordinate when showing relaxed open-mouth display (*n*=72)

Dominant individuals showed and did not show relaxed open-mouth behavior when approaching subordinates 47 times and 25 times, respectively. Dominant individuals were able to get closer to subordinates when they exhibited this display behavior (*t*=7.924, *P*<0.001) (Figure 5).

***: *P*<0.001.

## DISCUSSION

We explored four hypotheses related to relaxed open-mouth display within the OMUs of free-ranging *R. roxellana*. Results suggested that relaxed open-mouth display served four social functions in *R. roxellana* societies: that is, submission, reconciliation, affiliation and reassurance. Furthermore, an exchange of relaxed open-mouth display facilitated more significant outcomes than when one individual exhibited the behavior alone.

In our study group, relaxed open-mouth display usually functioned as a sign of submission. For subordinates, relaxed open-mouth display significantly reduced the probability of being displaced when dominant individuals approached (Table 2). For victims, relaxed open-mouth display significantly reduced the occurrence of continued aggression (Table 3). These outcomes are similar to those reported in previous study on *R. roxellana* populations in the Qinling Mountains of China, where adult females and juveniles were observed to open their mouths to exhibit submission, prevent aggression and maintain OMU stability (Yang et al., 2013).

Relaxed open-mouth display also served as a clear signal of reconciliation as aggression was unlikely to continue while aggressors and victims could reengage in social interactions in close proximity (Figure 4). These results are in agreement with those reported from a captive group of *R. roxellana* (Ren et al., 1991), as well as another colobine species such as *Trachypithecus francoisi* (Wang, 2009). This behavioral pattern can be explained as a mechanism of reconciliation, thus supporting the hypothesis of “increasing social tolerance” to pacify tension within groups (Aureli & van Schaik, 1991; de Waal & Ren, 1988), ease anxiety (Cords, 1992; Das et al., 1998), reduce continued aggression (Call et al., 1999; Cords, 1992; de Waal, 1993) and decrease heart rates (Smucny et al., 1996).

Similar to earlier descriptions (Ren et al., 1990b), relaxed open-mouth display in *R. roxellana* also had an affiliative effect, allowing subordinates to approach dominant individuals more closely (Figure 3). Interestingly, we noted that most individuals in the study group also frequently exhibited relaxed open-mouth display toward reserve staff during provisioning, suggesting possible affiliation and acceptance for easier food acquisition.

We also found that relaxed open-mouth display functioned as reassurance–a kind of affiliative signal displayed by dominant individuals towards subordinates to initiate closer proximity to each other (van Hooff, 1967; Wiper & Semple, 2007) (Figure 5). Dominant individuals can use this behavior to reassure and ease anxiety in subordinates who may treat the former as a potential threat (Cords, 1992; Das et al., 1998; Ren et al., 1990b). This behavior has also been reported when adult females care for their infants, when adult males or females solicit sexual partners for copulation, and when adult males pacify their own OMUs after conflict between two or more adults (Ren et al., 1990b). Thus, reassurance behavior may be instigated to reassure infants, sexual partners and unit members to gain fitness, social stability and self-assurance.

Within OMUs of *R. roxellana*, the exchange of relaxed open-mouth display between two individuals yielded more significant outcomes than when one individual displayed the behavior alone, allowing subordinates to approach dominant individuals for social activities such as sitting together, grooming and hugging (Figure 3), as well as reducing the distance between victims and aggressors after aggression (Bout & Thierry, 2005; van Hooff, 1967) (Figure 4). These findings are similar to observations in other non-human primates (Aureli, 1997; Bout & Thierry, 2005; Call et al., 1999; de Waal, 1986; de Waal & Luttrell, 1989; de Waal & Ren, 1988; de Waal & van Roosmalen, 1979; Wang, 2009; Wiper & Semple, 2007). Thus, *R. roxellana* appears to be a relatively mild and peaceful species with high intra-unit tolerance (Li et al., 2006; Wiper & Semple, 2007), and the exchange of relaxed open-mouth display helps maintain the stability and cohesion of the species’ multi-level social organization (Qi et al., 2014).

We quantitatively analyzed the functions and hypotheses of relaxed open-mouth display within OMUs of free-ranging *R. roxellana*, which are essential for understanding the social dynamics and mechanisms of multi-level societies.
